# Reduced Na^+^ and higher K^+^ channel expression and function contribute to right ventricular origin of arrhythmias in *Scn5a+/−* mice

**DOI:** 10.1098/rsob.120072

**Published:** 2012-06

**Authors:** Claire A. Martin, Urszula Siedlecka, Kristin Kemmerich, Jason Lawrence, James Cartledge, Laila Guzadhur, Nicola Brice, Andrew A. Grace, Christof Schwiening, Cesare M. Terracciano, Christopher L.-H. Huang

**Affiliations:** 1 Physiological Laboratory, University of Cambridge, Downing Site, Cambridge CB2 3EG, UK; 2 Laboratory of Cell Electrophysiology, Heart Science Centre, National Heart and Lung Institute, Imperial College London, Harefield Hospital, Harefield, Middlesex UB9 6JH, UK; 3 Laboratory of Molecular Biology, Medical Research Council, Hills Road, Cambridge CB2 0QH, UK; 4 Takeda Cambridge Limited, Cambridge Science Park, Cambridge CB4 0PA, UK; 5 Department of Biochemistry, University of Cambridge, Downing Site, Cambridge CB2 1QW, UK

**Keywords:** sodium channel, potassium channel, arrhythmia mechanisms, animal models, Brugada syndrome

## Abstract

Brugada syndrome (BrS) is associated with ventricular tachycardia originating particularly in the right ventricle (RV). We explore electrophysiological features predisposing to such arrhythmic tendency and their possible RV localization in a heterozygotic *Scn5a+/−* murine model. Na_v_1.5 mRNA and protein expression were lower in *Scn5a+/−* than wild-type (WT), with a further reduction in the RV compared with the left ventricle (LV). RVs showed higher expression levels of K_v_4.2, K_v_4.3 and KChIP2 in both *Scn5a+/−* and WT. Action potential upstroke velocity and maximum Na^+^ current (*I*_Na_) density were correspondingly decreased in *Scn5a+/−*, with a further reduction in the RV. The voltage dependence of inactivation was shifted to more negative values in *Scn5a+/−.* These findings are predictive of a localized depolarization abnormality leading to slowed conduction. Persistent Na^+^ current (*I*_pNa_) density was decreased in a similar pattern to *I*_Na_. RV transient outward current (*I*_to_) density was greater than LV in both WT and *Scn5a+/−*, and had larger time constants of inactivation. These findings were also consistent with the observation that AP durations were smallest in the RV of *Scn5a+/−*, fulfilling predictions of an increased heterogeneity of repolarization as an additional possible electrophysiological mechanism for arrhythmogenesis in BrS.

## 2. Introduction

Brugada syndrome is characterized by increased clinical risks of ventricular tachycardia (VT). It has been associated with a loss of Na^+^ channel function. Clinical studies suggest that BrS is an RV disease resulting in an electrocardiographic, right precordial, ST elevation, right bundle branch block and changes specific to RV epicardial AP waveforms [1]. A range of mechanisms may contribute to this arrhythmic phenotype. In addition to potential contributions from fibrotic change and reduced connexin expression [2], electrophysiological abnormalities could result in either abnormal depolarization [3] or repolarization [4]. For example, reduced *I*_Na_ could decrease conduction velocity. Abnormal Na^+^ expression and function may also affect action potential (AP) duration: while the transient Na^+^ current inactivates within a few milliseconds, a slowly inactivating component, *I*_pNa,_ could potentially play an important role in AP repolarization [5]. Although small, its persistence over the time course of an AP could transfer quantities of charge comparable with those carried by the transient current [5]. Finally, repolarization abnormalities could also result from reductions in the *I*_pNa_ relative to the early repolarizing *I*_to_, with or without accompanying changes in *I*_to_.

The present study tests a prediction that one or more of the latter electrophysiological abnormalities might be preferentially localized to the RV at the cellular and molecular level, and thereby give rise to the arrhythmic phenotype seen at the clinical or whole heart level. It thus explores for preferential alterations in *I*_Na_ and *I*_K_ expression in the RV in a murine heterozygotic *Scn5a*+/− model for BrS. Previous studies have either relied on largely non-invasive experiments in humans or on a canine pharmacological wedge preparation in which the BrS phenotype was replicated by multiple pharmacological interventions. In contrast, although BrS is a genetically heterogeneous condition, up to 30 per cent of patients have mutations in the *SCN5A* gene [6]. Thus far, it is the only gene that has been extensively studied in connection with BrS. Furthermore, the mouse model closely reproduces many of the key human features of BrS, including ST elevation [7], and both slowed conduction and repolarization heterogeneities leading to ventricular arrhythmogenesis originating in the RV, as demonstrated in mapping experiments [8]. It also shows the complex range of further phenotypes that include sick sinus syndrome and progressive conduction disorders associated with the clinical *SCN5A* gene modification [9]. Finally, *Scn5a+/−* hearts show fibrosis and reduced connexin expression that worsens with age [2], in line with similar clinical findings [10]. This appears to affect the RV to a greater extent than the LV, leading to a greater degree of conduction slowing in the RV and thus possibly contributing to the predisposition of the RV to re-entrant arrhythmias [3].

However, despite a detailed description of the electrophysiological abnormalities in the *Scn5a+/−* mice, so far no direct link has been made between the loss of Na^+^ channel function and the predilection for arrhythmias to be initiated in the RV. The only proved effective treatment of BrS so far is an implantable cardioverter–defibrillator, which is limited by low appropriate shock rates and device-related complications [11]. Further clarification of the relationship between arrhythmias and their basis in ion channel properties and localization could prove crucial in planning possible new pharmacological therapies for BrS. The present experiments investigate the relative expression and function of Na^+^ and K^+^ channels in the LV and RV of WT and *Scn5a*+/− hearts for the first time, and hence assess their contributions to conduction slowing and repolarization heterogeneities in the arrhythmogenic mechanism of BrS.

## 3. Methods

### 3.1. Experimental animals

Mice aged three to five months were obtained from breeding pairs of heterozygote *Scn5a*+/− and WT inbred 129/sv mice. Hearts from all groups were subject to identical experimental procedures, permitting their comparison. WT mice were initially supplied by Harlan (UK), and *Scn5a*+/− mice were originally generated from these as described previously [12]. Mice were killed by cervical dislocation, in compliance with the UK Animals (Scientific Procedures) Act 1986. The investigation conforms to the Guide for the Care and Use of Laboratory Animals and was performed under licences approved by local ethical committees and issued by the UK Home Office.

### 3.2. Real time reverse transcriptase-PCR

To quantify changes in the mRNA expression levels of Na^+^ channel α-subunits and a range of K^+^ channels in murine hearts, real time reverse transcriptase-PCR (RT-PCR) experiments were performed on an ABI 7500 Fast Cycler (Applied Biosystems, Warrington, UK). Total RNA was isolated from the LV and RV of WT and *Scn5a*+/− mice (*n* = 4 each) using a Qiagen RNAeasy kit. Excised tissues were stored in RNAlater (Ambion, Warrington, UK) to maintain the integrity of the RNA before isolation. The total RNA was reverse transcribed into cDNA using random hexamer primers and a SuperScript III kit (Invitrogen, Paisley, UK). Oligos for *Scn5a* were fluorescein amidite (FAM) labelled (Applied Biosystems). All experiments were performed in duplicate.

The number of copies of mRNA was calculated from its respective threshold cycle (C_T_) using a standard curve. The 2^−*ΔΔ*CT^ method [13] was used to calculate gene expression relative to the expression of the housekeeper gene, glyceraldehyde-3-phosphate dehydrogenase (GAPDH). These values were then normalized to the mean Na_v_1.5 gene expression in the LV of the WT mice.

### 3.3. Western blots

Hearts were excised and placed in ice-cold Krebs–Henseleit buffer containing: 119 mM NaCl, 25 mM NaHCO_3_, 4 mM KCl, 1.2 mM KH_2_PO_4_, 1 mM MgCl_2_, 1.8 mM CaCl_2_, 10 mM glucose and 2 mM sodium pyruvate (pH 7.4). The ventricles were removed and the LV and RV free walls were isolated and flash-frozen. The tissues were ground in liquid nitrogen with a pestle and mortar. They were then homogenized using a Dounce homogenizer (Jencons, Lutterworth, UK) in ice-cold lysis buffer containing: 50 mM Tris pH 8.0, 150 mM NaCl, 1 per cent NP40, 0.2 per cent SDS, 0.5 per cent Na Deoxycholate, 1 mM EDTA and complete protease inhibitor cocktail (Roche, Burgess Hill, UK), and rotated for 1 h at 4°C. The samples were then centrifuged at 2000*g* for 10 min and the supernatant kept. The pellet was resuspended in lysis buffer and recentrifuged at 2000*g* for a further 10 min, and the second supernatant added to the first. The combined supernatants were then ultracentrifuged at 100 000*g* for 1 h, and the pellet suspended in buffer containing: 4 mM HEPES, 320 mM sucrose and complete protease inhibitor cocktail. The protein concentration of each sample was established using a Pierce bicinchoninic acid assay kit (Thermo Scientific, Rockford, USA), using microplate assays in triplicate.

All equipment and consumables mentioned in this paragraph are from Invitrogen unless otherwise stated. Protein samples were mixed with 4x NuPAGE LDS sample buffer, 10x β-mercaptoethanol and H_2_O, and were heated at 70°C for 10 min. XCell SureLock Mini-Cells were used to run gel electrophoresis. One of two sets of electrophoresis conditions were used depending on whether the protein to be analysed was of high or low molecular weight. For high molecular weight proteins, electrophoresis was carried out using NuPAGE Novex 3 to 8 per cent Tris-Acetate gels at 30 V for 90 min, and then at 140 V overnight at 4°C with NuPAGE Tris-Acetate running buffer and NuPAGE antioxidant, alongside a Hi-Mark pre-stained high-molecular-weight protein standard. For low-molecular-weight proteins, electrophoresis was carried out using NuPAGE Novex 4 to 12 per cent Bis-Tris gels at 140 V for 2 h with NuPAGE MES SDS running buffer and NuPAGE antioxidant, alongside a pre-stained protein standard. Twenty mM of total protein was loaded in each lane. Protein bands were transferred onto polyvinylidene difluoride (PVDF) membranes by an XCell II Blot Module, with NuPAGE transfer buffer, 10 per cent methanol and NuPAGE antioxidant, at 100 mA overnight at 4°C. Membranes were blocked for 5 h in 5 per cent BSA-phosphate-buffered saline (PBS)—1 per cent Tween, prior to overnight incubation at 4°C with primary antibody. Antibodies used were to Na_v_1.5 (1:500, ASC005, Caltag Medisystems, Alomone, Israel), and K_v_1.5, K_v_4.2, K_v_4.3, K_ir_2.1, K_v_1.4 and KChIP2 (1:1000, Abcam, UK). The membranes were incubated for 1 h at room temperature with secondary antibody conjugated with horseradish peroxidase from Sigma-Aldrich (Poole, Dorset, UK). Western blot development was performed with Amersham ECL-plus reagents (Amersham Biosciences, Amersham, UK).

To confirm equal protein loading, the PVDF membranes were stripped by incubation in stripping buffer (200 mM glycine, 1% SDS, 1% Tween, adjusted to pH 2.2) and then rinsed first with PBS and then with PBS-1 per cent Tween. The membranes were then reblocked, and incubated overnight with antibody to either heavy chain cardiac myosin (1:200, ab50967, Abcam, Cambridge, UK) for high-molecular weight-proteins, or to GAPDH (1:5000, ab9482, Abcam) for low-molecular-weight proteins. Secondary antibody incubation and detection were carried out as above. Radiographs were scanned and areas of interest cropped. Band intensity was calculated using ImageJ (NIH, Bethesda, USA), and plots drawn of protein expression normalized to either heavy chain myosin or GAPDH expression.

### 3.4. Ventricular myocyte isolation

Myocytes were isolated as previously described [14]. Mouse hearts were rapidly removed and placed in ice-cold normal Tyrode (NT) solution containing: 140 mM NaCl, 6 mM KCl, 1 mM MgCl_2_, 1 mM CaCl_2_, 10 mM glucose, 10 mM HEPES, pH adjusted to 7.4 with 2 M NaOH. The ascending aorta was cannulated with a blunted 21 g needle. The heart was then retrogradely perfused for 2–3 min at 37°C with NT solution followed by a further 5 min in low Ca^2+^ (LC) solution containing: 120 mM NaCl, 5.4 mM KCl, 5 mM MgSO_4_, 0.045 mM CaCl_2_, 5 mM sodium pyruvate, 20 mM glucose, 20 mM taurine, 10 mM HEPES, 5 mM nitrilotriacetic acid, bubbled with 100% O_2_; pH 6.96. The solution was changed to an enzyme solution (ES) containing: 120 mM NaCl, 5.4 mM KCl, 5 mM MgSO_4_, 0.2 mM CaCl_2_, 5 mM sodium pyruvate, 20 mM glucose, 20 mM taurine, 10 mM HEPES, bubbled with 100% O_2_; pH 7.4 with addition of protease (4 U ml^−1^, Sigma) for 1 min, followed by perfusion for 8 min with ES solution containing collagenase (0.8 mg ml^−1^; Worthington) and hyaluronidase (0.5 mg ml^−1^, Sigma). At this stage, the heart was swollen and flaccid but with anatomy still intact. The free walls of the LV and RV were then separated and cut into small pieces using a scalpel blade. The tissue was then agitated for a further 5 min in ES solution. This suspension was gently triturated using a pipette, filtered and resuspended in ES solution. Myocytes were stored in ES solution at room temperature and used within 7–8 h of isolation. Isolated myocytes were checked under a light microscope to confirm cell integrity. Live myocytes appeared as smooth and rod-shaped cells.

### 3.5. Electrophysiology measurements

#### 3.5.1. Measurement of action potentials

Cells were superfused with NT solution at 37°C and studied using a perforated patch technique using an Axon 2B amplifier (Axon Instruments, CA, USA). The pipette resistance was 1–2 M*Ω*, and the pipette-filling solution contained: 125 mM KCH_3_O_3_S, 20 mM KCl, 10 mM NaCl, 10 mM HEPES, 5 mM MgCl_2_, pH 7.2 with KOH, plus amphotericin-B (final concentration 240 μg ml^−1^). APs were measured in current-clamp mode after stimulation at 1, 3 and 5 Hz using a 1 ms, 1.2–1.4 nA pulse. The APs measured were analysed using pClamp v. 10 software (Molecular Devices, USA), and averaged over 30 consecutive beats. Traces were then averaged with reference to the stimulation signal, and times to 50 per cent (APD_50_), 70 per cent (APD_70_) and 90 per cent repolarization (APD_90_) were taken as a measure of the AP duration for comparison between groups. Maximum upstroke velocity was measured as the maximum gradient of the upstroke of the AP waveform.

#### 3.5.2. Current density measurements

For all current density measurements, the whole-cell configuration of the patch-clamping technique was employed using a Multiclamp 700A amplifier (Molecular Devices). Borosilicate glass pipettes (0.86 mm outer diameter; Harvard Apparatus, Kent, UK) were used, with a resistance of 1.5–2.5 M*Ω*. All the experiments were performed at 37°C, apart from those measuring the fast Na^+^ current, which were performed at a constant room temperature of 22°C. The rate of superfusion was 2–3 ml min^−1^. Miniature solenoid valves (The Lee Company, Essex, UK) were used to produce fast changes in superfusate. All the recordings were done after 3 min of membrane rupture, after which time pilot experiments demonstrated stable voltage dependency of currents. Each set of measurements took approximately 5 min to complete. Capacitance and 80 to 90 per cent series resistance compensation was performed prior to each recording. Only cells with initial resting potentials between −70 and −80 mV, initial seal resistances greater than 5 G*Ω* with stable residual series resistances less than 1 M*Ω*, and low leakage currents (less than 0.1 nA at −120 mV) were studied. Cell stability in the course of the experiments was further followed by comparing transients from the same (largest) voltage steps at successive times during the protocol.

The currents obtained were filtered at a 10 kHz high–frequency cut-off (four pole Bessel filter) and digitized at 100 kHz using pClamp software.

The membrane capacitance (in picofarad) of each cell was initially measured by integrating the area under a capacitative transient induced by a 10 mV hyperpolarizing clamp step (from −80 to −90 mV) and dividing this area by the voltage step. There were no significant differences in mean cell capacitance between groups (122.4 ± 6 pF in WT LV, 119.5 ± 7 pF in WT RV, 129.3 ± 6 pF in *Scn5a*+/− LV and 126.6 ± 9 pF in *Scn5a*+/− RV; *n* = 36; *p* > 0.05). All the currents were then normalized to the whole-cell capacitance for cross-group comparisons.

### 3.6. Na^+^ current, *I*_Na_

The fast Na^+^ current *I*_Na_ was measured using a technique previously described [15]. Pipettes were filled with: 70.26 mM CsCl, 24.74 mM Cs-aspartate, 10 mM HEPES, 1 mM Na_2_ATP, 4 mM MgATP, 1.37 mM MgCl_2_ and 10 mM Cs-BAPTA, adjusted to pH 7.2 using CsOH. Cells were initially perfused with NT solution containing 200 μM Cd^2+^. To achieve optimum voltage control, Na^+^ currents were recorded in a low [Na^+^] solution. This solution contained: 10 mM NaCl, 140 mM CsCl, 10 mM glucose, 10 mM HEPES, 1 mM MgCl_2_ and 1 mM CaCl_2_, adjusted to pH 7.4 using CsOH. Na^+^ currents were initially measured before and after perfusion with 10 μM tetrodotoxin (TTX), and the TTX sensitive current calculated; however, pilot studies showed that under our experimental conditions, TTX eliminated the whole current being studied, and therefore showed that we were specifically recording the Na^+^ current before addition of TTX (figure 4). Capacitance subtraction was not performed and thus recordings demonstrated an initial spike; however, this was fast enough not to interfere with the subsequent Na^+^ current and the pilot studies using TTX subtraction eliminated the spike, thus demonstrating that it was due entirely to the capacitative transient.

The currents obtained were filtered at a 10 kHz high-frequency cut off and digitized at 100 kHz using pClamp software. In each experiment, the series resistance was compensated by 80 to 90 per cent and each protocol used a holding potential of −120 mV. The equilibrium potential for Na^+^ was calculated from the Nernst equation to be +40 mV, which was confirmed through initial experiments recording voltage steps to this level. For fast Na^+^ current recordings, the mean values of residual series resistance after 80 to 90 per cent compensation were 0.53 ± 0.1 M*Ω* for WT LV, 0.49 ± 0.1 M*Ω* for WT RV, 0.64 ± 0.1 M*Ω* for *Scn5a*+/− LV and 0.67 ± 0.1 M*Ω* for *Scn5a*+/− RV. The current required to keep the cells at holding potential was 0.2 ± 0.01 nA, resulting in a voltage error at the holding potential less than 0.1 mV. The peak values of current flowing during the voltage steps were 5.4 ± 0.1 nA for WT LV, 5.3 ± 0.1 nA for WT RV, 4.1 ± 0.1 nA for *Scn5a*+/− LV and 2.7 ± 0.1 nA for *Scn5a* +/− RV. Thus, peak voltage error resulting from residual series resistance was 2.9 ± 0.1 mV for WT LV, 2.6 ± 0.1 mV for WT RV, 2.6 ± 0.1 mV for *Scn5a*+/− LV and 1.8 ± 0.1 mV for *Scn5a*+/− RV. If experiments demonstrated evidence of inadequate voltage control (e.g. a ‘threshold phenomenon’ near the voltage range for Na*^+^* channel activation) data were discarded. The absence of crossover of the current traces as the current magnitude was changed and the shape of the current–voltage (*I*–*V*) relationship remaining unchanged for difference peak current values further suggest adequate voltage control [16,17].

First, the voltage dependence of activation was studied by applying depolarizing pulses of 250 ms duration over a series of voltage steps from a fixed holding voltage of −120 mV, in 5 mV increments, to +20 mV. The peak current densities (pA/pF) were calculated by dividing the peak currents amplitude by C_m_ and the *I*–*V* relationship plotted. The values of peak Na^+^ conductance (*g*_Na_) were determined from the equation

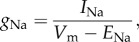

as first described by Hodgkin *et al*. [18] as a linear approximation of the Goldman–Hodgkin–Katz equation and subsequently used extensively in patch clamp analysis of cardiac myocytes [15,19–21], where *I*_Na_ is the peak current density and the *E*_Na_ is the [Na^+^] reversal potential. The *g*_Na_ values calculated from each myocyte were normalized against the corresponding peak *g*_Na_ value. When plotted against the test-pulse voltage, the data assumed a sigmoid function, increasing with membrane potential.

The voltage dependence of steady-state inactivation was also examined using conditioning pre-pulses of 500 ms duration. These variable pre-pulses were applied over a voltage range from −140 to −30 in 5 mV increments. Each pre-pulse was followed by a constant test pulse to −20 mV at a duration of 20 ms. Normalized currents were then plotted against the pre-pulse voltage. The data assumed a sigmoid function, decreasing with membrane potential. Both inactivation and activation data were fitted with a single Boltzmann function, as used extensively in previous studies [15,20–24], of the form:

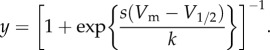



For activation curves, the *y*-values correspond to the normalized conductance values and constant *s* equals −1. For inactivation curves, the *y*-values correspond to the normalized current values and constant *s* equals +1. This yields the half-maximal voltage (*V*_1/2_) and exponential slope factor (*k*) values.

#### 3.6.1. Persistent Na^+^ current

The persistent Na^+^ current (*I*_pNa_) was measured using a technique previously described [25]. Cells were superfused at 37°C with modified NT solution, with K^+^ substituted with Cs^+^, and containing 10 μM nifedipine (Sigma, Poole, UK), 100 μM strophantidin (Sigma) and 30 μM niflumic acid (Sigma). The pipette filling solution contained: 115 mM Cs-aspartate, 20 mM TEA-Cl, 10 mM EGTA, 10 mM HEPES, 5 mM MgATP, pH 7.2. From a holding potential of −100 mV, increasing voltage (20 mV) steps from −80 mV to +20 mV for 1 s were applied with a cycle length of 5 s. The protocol was repeated in the presence of 20 μM TTX (Alomone Labs., Israel), and the TTX-sensitive current was analysed. *I*_pNa_ density was taken as the average current between 30 and 80 ms after depolarization and normalized to cell capacitance. This period was chosen because this is approximately the range of APDs measured in our experiments.

#### 3.6.2. Transient outward current, *I*_to_

The transient outward current *I*_to_ was measured using a technique previously described [26]. Cells were superfused with NT solution containing 200 μM Cd^2+^ and the pipette-filling solution contained: 120 mM K-glutamate, 10 mM KCl, 2 mM MgCl_2_, 10 mM HEPES, 5 mM EGTA, 2 mM Mg-ATP, pH 7.2 with KOH at 37°C. Pipette resistance was 1.5–2.5 M*Ω*. *I*_to_ was elicited by increasing voltage steps (1 s duration) from −40 mV to +70 mV with a cycle length of 5 s in increments of 10 mV. In these studies, the transient outward current was separated from the other currents by the following procedures. The holding potential was held at −40 mV to inactivate the sodium current. *I*_Ca_ was largely blocked by 0.2 mM CdCl_2_ included in the recording solution. EGTA was included in the pipette solution to minimize Ca^2+^-induced inactivation. *I*_to_ was taken as the peak of the transient outward current *I*_to,peak_ from which the steady-state current *I*_ss_ had been subtracted. While *I*_Ks_ may also demonstrate inactivation, studies have shown that this plays a relatively minor role in repolarization in the murine ventricle [27,28]. As for *I*_Na_*,* the *I*–*V* relationship was plotted, as well as the voltage dependence of activation as determined from conductance calculations, and fitted with a single Boltzmann distribution. The time course of inactivation of the decay phase of the transient outward current was best fit by a double exponential decay function in the following form:

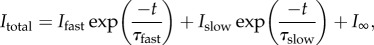



The two time constants were plotted as a function of voltage. Steady-state inactivation parameters of *I*_to,peak_ were obtained with a double-pulse protocol using 1 s prepulses to voltages from −100 to +20 mV from a holding potential of −100 mV. Peak currents at the test pulse to +40 mV were normalized to the maximal transient outward current elicited by the test pulse and fitted by a single Boltzmann distribution.

### 3.7. Statistical procedures

Patch clamp measurements were made from a set of 8 WT and 8 *Scn5a*+/− mice, with *n* numbers representing the total number of cells studied. 4 WT and 4 *Scn5a*+/− mice were each used for RT-PCR experiments and a further four each for Western blot experiments. All electrophysiological parameters and expression levels of protein and mRNA were expressed as mean ± s.e.m. values. Their significant differences were analysed using ANOVA applied to all groups with post-hoc Tukey's honestly significant difference tests then applied to paired groups. A *p*-value of less than 0.05 was the criterion for significance.

## 4. Results

### 4.1. Real-time reverse transcriptase-PCR

**Figure 1.**
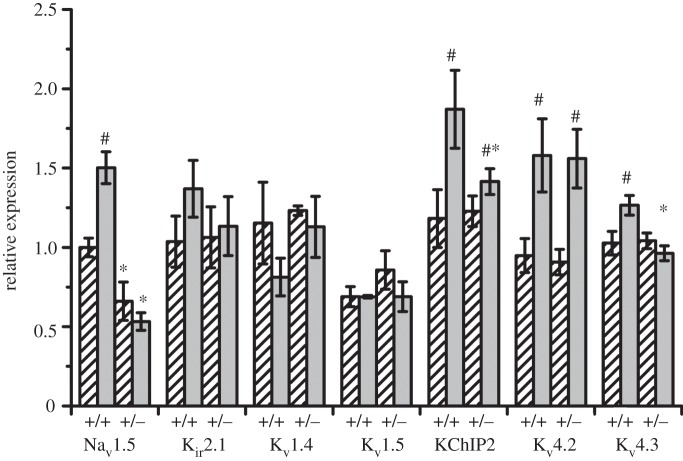
mRNA expression patterns. Graph of mRNA expression in Na^+^ and K^+^ channels in the LV (bars with stripes) and RV (grey bars) of WT and *Scn5a*+/− hearts. Values are given relative to Na_v_1.5 mRNA expression in LV WT hearts. Significant differences: asterisks (*), effect of genotype; hashes (#), effect of cardiac ventricle (*n* = 4 for each group).

Na_v_1.5 mRNA expression was significantly lower in *Scn5a*+/− hearts than in WT, as previously reported [22] (figure 1). While this was true in both the LV and RV, there was a bigger drop in the RV. While in the LV expression fell to approximately two-thirds of that in the WT, in the RV it fell to about one-third of that in the WT. Interestingly, in the WT there was significantly higher expression of Na_v_1.5 mRNA in the RV than LV, while in the *Scn5a*+/− hearts, there was no significant difference between mRNA expression in the two ventricles.

Regarding mRNA expression for K^+^ channels in the heart, we found no significant differences either between LV and RV, or between WT and *Scn5a*+/− hearts for K_ir_2.1, K_v_1.4 and K_v_1.5. However, there were significantly higher mRNA expression levels of K_v_4.2 in RVs in both WT and *Scn5a*+/− hearts, although there was no difference between WT and *Scn5a*+/−. K_v_4.3 showed a small increase in WT RV compared with the WT LV or *Scn5a*+/− RV, but there were no differences between LV WT or *Scn5a*+/−, nor between LV and RV *Scn5a*+/−. Lastly, there was higher mRNA expression of KChIP2 in WT than *Scn5a*+/− RV, and in the RV than LV, of both WT and *Scn5a*+/− hearts.

### 4.2. Western blots

**Figure 2.**
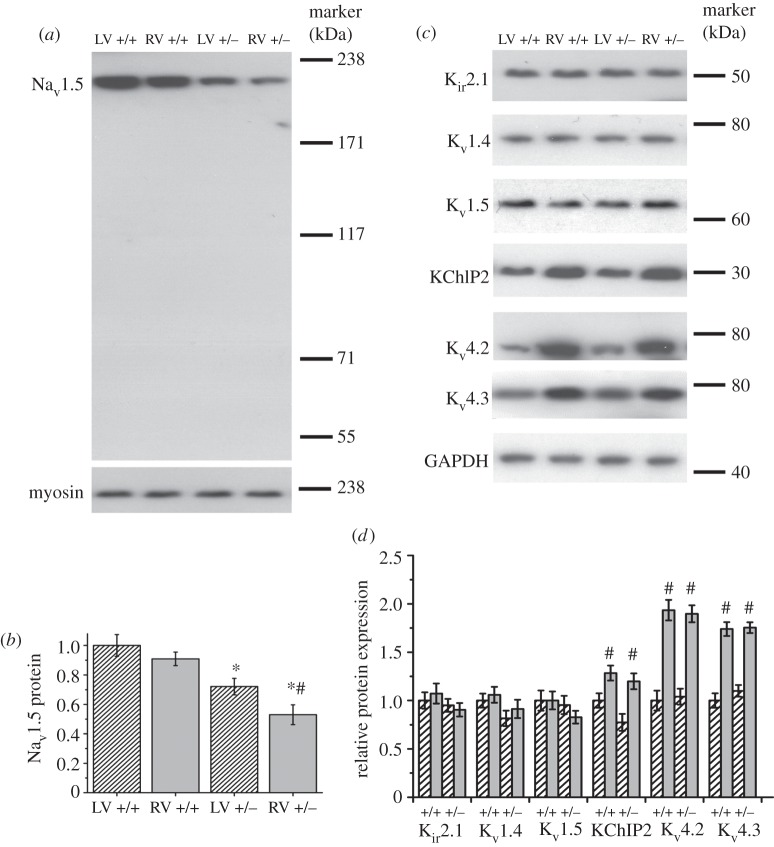
Protein expression patterns. (*a*) Western blot using anti-Na_v_1.5 antibody on extracts from LV (bars with stripes) and RV (grey bars) of WT and *Scn5a*+/− hearts, using anti-heavy chain myosin antibody as a loading control. The whole lane is shown to demonstrate the single band. (*b*) Graph of Na_v_1.5 expression levels relative to expression levels in LV WT, calculated from densitometric analysis of protein expression with band intensity normalized to anti-myosin expression. (*c*) Representative Western blots of antibody-specific binding to a range of K^+^ channels and to glyceraldehyde-3-phosphate dehydrogenase used as a loading control. (*d*) Graph of K^+^ channel expression levels relative to expression levels in LV WT. Significant differences: asterisks (*), effect of genotype; hashes (#) effect of cardiac ventricle (*n* = 4 for each group).

Western blots of Na_v_1.5 demonstrated a single band at around 220 kDa (predicted weight 227 kDa; figure 2*a*). There was no significant difference in the Na_v_1.5 expression found between the LV and RV of WT hearts (figure 2*b*). As expected, *Scn5a*+/− hearts had significantly reduced Na_v_1.5 expression, but there was a further difference between LV and RV, with approximately 30 per cent reduction in the LV and approximately 50 per cent reduction in the RV when compared with WT, resulting in a significantly lower expression of Na_v_1.5 in the RV compared with the LV of *Scn5a*+/− hearts. For K^+^ channels, there were no significant differences in protein expression either between LV and RV or WT and *Scn5a*+/− hearts for K_ir_2.1, K_v_1.4 or K_v_1.5 (figure 2*c,d*). However, there were significantly higher protein levels in the RV than LV for both WT and *Scn5a*+/− hearts for K_v_4.2, K_v_4.3 and KChIP2, with no difference between WT and *Scn5a*+/−.

### 4.3. Electrophysiology

APDs and *I*_Na_, *I*_pNa_ and *I*_to_ densities were measured using patch clamp techniques. APs from all groups demonstrated a triangular AP morphology, with a resting membrane potential of around −70 mV and with an overshoot to around +40 mV, in agreement with previous studies in WT mice [29].

### 4.4. *Scn5a*+/− right ventricle action potentials have shorter durations and lower maximum upstroke velocities compared with respective left ventricles and with wild-type

**Figure 3.**
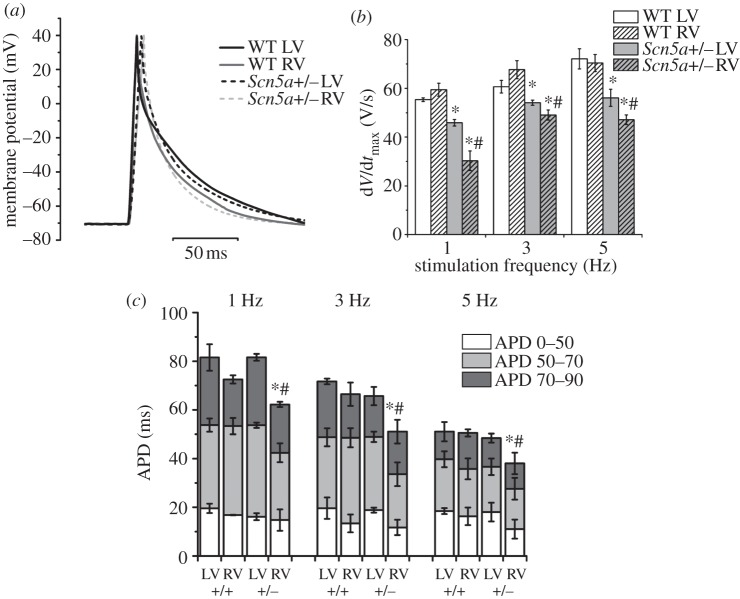
AP measurements. (*a*) Examples of AP traces at 1 Hz pacing overlaid from the LV and RV of WT and *Scn5a*+/− myocytes. (*b*) Bar chart comparing upstroke velocity. (*c*) Bar chart comparing APDs with APD_50_s, APD_70_s and APD_90_s superimposed. Significant differences: asterisks (*), effect of genotype; hashes (#), effect of cardiac ventricle (*n* = 20 for each group).

The maximum upstroke velocity (d*V*/d*t*_max_) of the APs, measured as the maximum gradient of the AP upstroke, can be used to give an indication of Na^+^ channel function [30]. d*V*/d*t*_max_ was reduced in *Scn5a*+/− hearts compared with WT in both LV and RV myocytes at all pacing frequencies (figure 3). The d*V*/d*t*_max_ values were not significantly different between LV and RV in WT myocytes, but were significantly reduced in RV compared with LV in *Scn5a*+/− myocytes. There were no significant differences overall in the amplitude of the APs between any group.

At all three pacing frequencies, APD_90_ was significantly lower in the RV compared with the LV of *Scn5a*+/− myocytes, but not in WT. The APD_90_ of RV *Scn5a*+/− myocytes was also significantly less than that of RV WT myocytes, while there were no differences between the respective LVs. Similar results were obtained for the APD_70_ measurements. These myocyte data correlate well with previous studies from APD recordings using monophasic AP electrodes in Langendorff perfused hearts [31].

### 4.5. *Scn5a*+/− right ventricle myocytes have lower *I*_Na_ than the respective left ventricle and in wild-type

**Figure 4.**
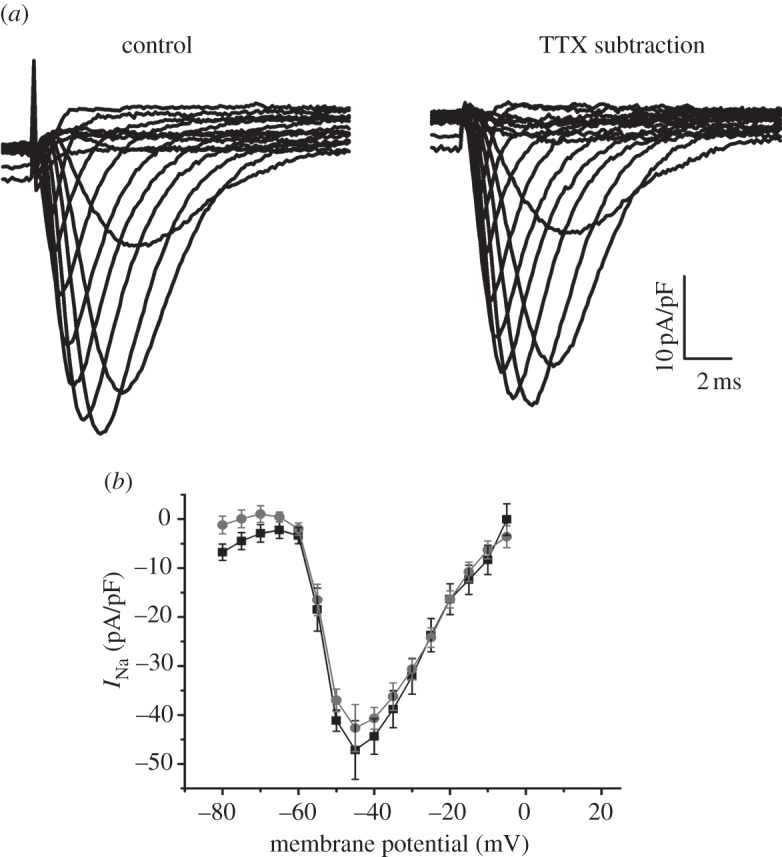
(*a*) Example trace of an *I*_Na_ current from a myocyte from the LV of a WT heart, before addition of 10 μM TTX, and then with TTX subtraction. (*b*) *I*–*V* curve for the two conditions (squares, control; circles, TTX substraction), with no significant differences (*n* = 4).

Na^+^ currents were initially measured before and after perfusion with 10 μM TTX, and the TTX-sensitive current calculated; however, pilot studies showed that under our experimental conditions, TTX eliminated the entire current being studied, and therefore showed that we were specifically recording the Na^+^ current without the need for TTX subtraction (figure 4). There were no significant differences in mean capacitance of myocytes between any of the groups.

**Figure 5.**
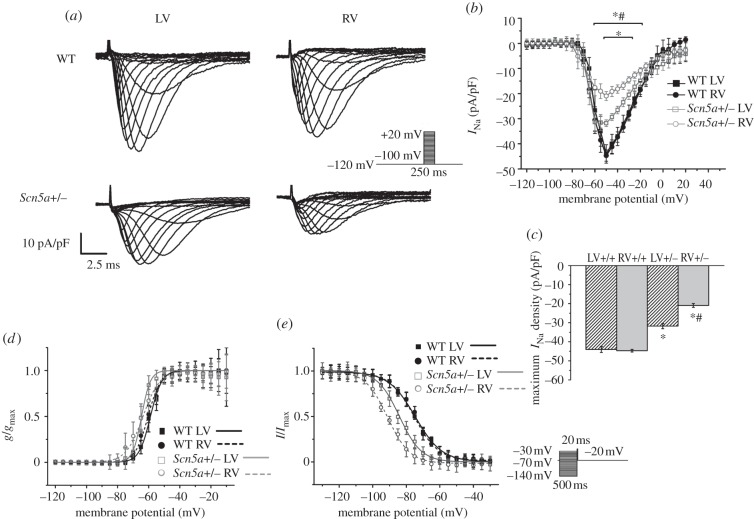
*I*_Na_ measurements. (*a*) Example traces of Na^+^ currents from myocytes from the LV and RV of WT and *Scn5a*+/− hearts. The traces have been normalized to cell capacitance, and the protocol is shown in the inset. (*b*) I–V relationship of Na^+^ current in myocytes from the four groups (*n* = 17). (*c*) Maximum *I*_Na_ density of each group (*n* = 17). (*d*) Activation and (*e*) inactivation curves in myocytes from the four groups, with Boltzmann fits (*n* = 17 for activation, *n* = 12 for inactivation). Significant differences: asterisks (*), effect of genotype; hashes (#) effect of cardiac ventricle. Note in (*b*), upper asterisk denotes differences in genotype for RV, and lower asterisk for LV.

Values of maximum Na^+^ current density (*I*_Na_max) from WT myocytes were in agreement with those from previous studies [23]. There were no significant differences in *I*_Na_max or *I*–*V* relationship between LV and RV myocytes from WT hearts (figure 5*a–c*). *I*_Na_max was significantly less in both LV and RV of *Scn5a*+/− hearts than in WT; this was also reflected in differences in the *I*–*V* relationship. In addition, in *Scn5a*+/− hearts Na^+^ current density was reduced in the RV compared with the LV. Overall, *I*_Na_max in the RV of *Scn5a*+/− hearts was around 50 per cent that of RV WT, while in the *Scn5a*+/− LV it was around 70 per cent of WT LV.

Activation curves yielded parameters of voltage dependence of activation similar to previous studies [12,22,23]. There were no significant differences in these parameters between any of the groups studied (figure 5*d* and table 1). The inactivation curve was shifted to more negative values in the *Scn5a*+/− hearts compared with WT for both RV and LV, and for the RV was further shifted to more negative values than the LV in *Scn5a*+/− hearts (figure 5*e* and table 1). Thus, the fraction of Na^+^ channels available for cardiac depolarization is smaller in the *Scn5a*+/− than WT*,* and further reduced in the RV.

**Table 1.** Parameters of activation and inactivation from Boltzmann fits (millivolts) to the curves produced from *I*_Na_ in myocytes from the LV and RV of WT and *Scn5a*+/− hearts (*n* = 17 for activation; *n* = 12 for inactivation for each group).

	activation		inactivation	
	*V*_1/2_	*k*	*V*_1/2_	*k*
WT LV	−58.88 ± 2.85	3.65 ± 0.66	−74.64 ± 1.83	7.95 ± 0.62
WT RV	−60.42 ± 3.04	4.14 ± 0.42	−75.35 ± 1.72	7.21 ± 0.60
*Scn5a*+/− LV	−64.61 ± 2.96	3.44 ± 0.78	−84.16 ± 1.53^a^	6.56 ± 0.71
*Scn5a*+/− RV	−63.69 ± 3.40	5.46 ± 0.93	−91.12 ± 1.64^a,b^	6.61 ± 0.74

^a^Significant effect of genotype.

^b^Significant effect of cardiac ventricle.

### 4.6. Lower *I*_pNa_ in *Scn5a*+/− right ventricle myocytes compared with respective left ventricle and with wild-type

**Figure 6.**
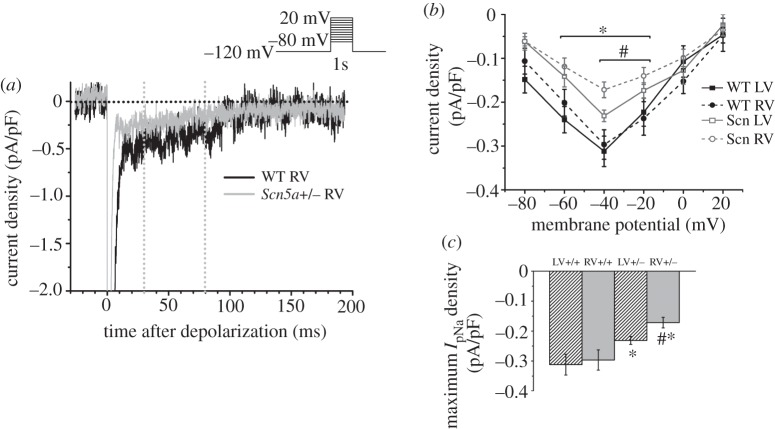
*I*_pNa_ measurements. (*a*) Example traces from a test pulse to −20 mV from the LV and RV of WT and *Scn5a*+/− myocytes. The vertical lines denote 30 and 80 ms after depolarization. (*b*) *I*–*V* relationship of mean *I*_pNa_ from 30 to 80 ms after depolarization in myocytes from each group. (*c*) Bar graph of maximum *I*_pNa_ density averaged from 30 to 80 ms after depolarization. Significant differences: asterisks (*), effect of genotype; hashes (#), effect of cardiac ventricle (*n* = 10 for each group).

We proceeded to measure the persistent Na^+^ current, *I*_pNa_. Following a similar pattern to *I*_Na_, maximum *I*_pNa_ was significantly smaller in *Scn5a*+/− than WT myocytes, and while there was no difference in maximum *I*_pNa_ between WT LV and RV, it was significantly smaller in the RV of *Scn5a*+/− hearts than LV (figure 6).

### 4.7. Higher *I*_to_ in right ventricle than left ventricle of wild-type and *Scn5a*+/− myocytes

**Figure 7.**
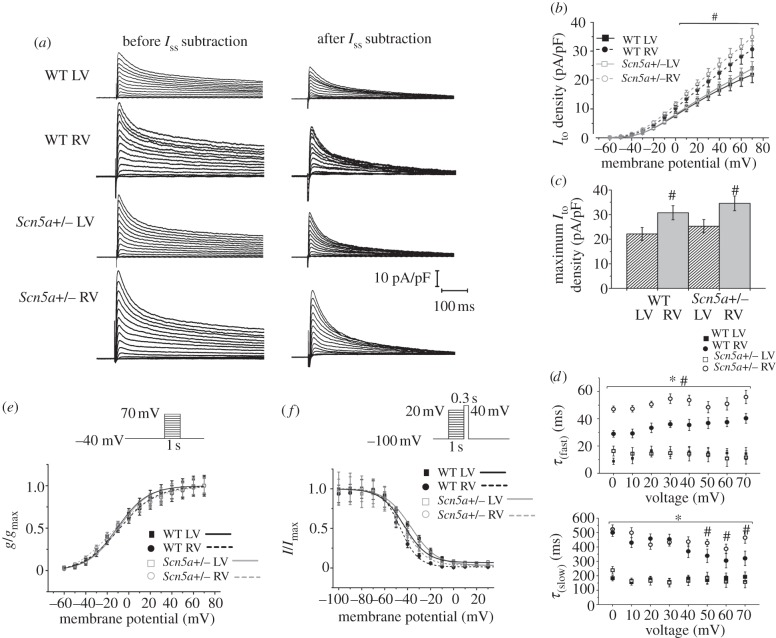
*I*_to_ measurements. (*a*) Typical raw trace from the LV of a WT heart and example traces from the LV and RV of WT and *Scn5a*+/− hearts where the sustained current has been subtracted from the point of depolarization onwards, thus leaving the transient outward current. The traces have been normalized to cell capacitance, and the protocol is shown in the inset. (*b*) I–V relationship of myocytes from the LV and RV of WT and *Scn5a*+/− hearts. (*c*) Bar graph of maximum *I*_to_ density. (*d*) Kinetics of inactivation from myocytes from all groups, with the curve fitted to a double exponential decay function. Significant differences: asterisks (*), effect of genotype; hashes (#), effect of cardiac ventricle (*n* = 14 for each group). (*e*) Activation and (*f*) inactivation curves from myocytes from the LV and RV of WT and *Scn5a*+/− hearts, with Boltzmann fits (*n* = 14 for activation, *n* = 13 for inactivation for each group).

Lastly, we measured inactivating K^+^ current by subtracting the sustained current *I*_ss_ from the point of depolarization onwards in raw traces (figure 7*a*). The inactivating K^+^ current was taken to represent *I*_to_ max, as, while more slowly inactivating K^+^ currents do exist in the mouse ventricle, they are relatively small [27]. There were no significant differences in *I*_ss_ amplitude between any of the groups. *I*_to_max was significantly greater in the RV than LV of both *Scn5a*+/− and WT, which was also reflected in differences in the I–V relationship (figure 7*b*,*c*). There were no significant differences in *I*_to_max or the *I*–*V* relationship between WT and *Scn5a*+/− myocytes from either LV or RV.

Analysis of the kinetics of inactivation through fitting to a double exponential decay function demonstrated three components of the K^+^ current, which are likely to represent *I*_to,_ *I*_Ks_ and *I*_ss_, respectively [32]. The amplitude of *I*_fast_ was approximately 10 times that of *I*_slow_, supporting evidence that *I*_to_ is the dominant inactivating K^+^ current in the mouse ventricle [27]. Figure 7*d* shows the time constants of decay of the two inactivating components from myocytes from all groups. There was no clear voltage dependence, similar to findings in other studies [32].

The RV showed values of *τ*_(fast)_ and *τ*_(slow)_ that were higher than their respective values in the LV in both the WT and *Scn5a*+/−. These time constants were then higher in the *Scn5a*+/− than WT in the RV for all voltages for *τ*_(fast)_ and at more positive voltages for *τ*_(slow)._ In contrast, the LV of both WT and *Scn5a*+/− showed similar values of *τ*_(fast)_ and *τ*_(slow)_.

There were no significant differences in voltage dependence of activation (figure 7*e* and table 2). Voltage dependence of inactivation was shifted to more negative values in the RV compared with LV of both WT and *Scn5a*+/− hearts, but there was a shift to more positive values for *Scn5a*+/− myocytes compared with WT (figure 7*f* and table 2).

**Table 2.** Parameters of activation and inactivation from Boltzmann fits (millivolts), and the time constants of inactivation from the double exponential fit to the curves produced from *I*_to_ in myocytes from the LV and RV of WT and *Scn5a*+/− hearts (*n* = 14 for each group).

	activation		inactivation	
	*V*_1/2_	*k*	*V*_1/2_	*k*
WT LV	−11.06 ± 2.54	14.26 ± 3.24	−42.72 ± 1.86	9.18 ± 1.10
WT RV	−9.07 ± 2.85	17.24 ± 3.21	−46.40 ± 1.72^a^	7.80 ± 0.92
*Scn5a*+/− LV	−9.58 ± 3.20	14.15 ± 3.95	−37.13 ± 0.91^b^	11.30 ± 0.70
*Scn5a*+/− RV	−15.57 ± 3.44	20.69 ± 3.55	−42.98 ± 1.01^a,b^	10.15 ± 0.73

^a^Significant effect of cardiac ventricle.

^b^Significant effect of genotype.

## 5. Discussion

These experiments use a *Scn5a*+/− murine model to test a hypothesis that localizes potentially arrhythmogenic electrophysiological mechanisms to the RV. This system has previously been used to model BrS, and mapping studies have demonstrated slowed conduction and repolarization heterogeneities localized to the RV leading to re-entrant polymorphic VT originating specifically in the RV [3,8]. Our findings add to (i) reports associating the *Scn5a*+/− mutation with reduced Na^+^ current expression [12], and (ii) previous studies in WT mice that demonstrated higher *Scn5a* mRNA and protein expression and Na^+^ channel availability in endocardium than epicardium [33]. Neither study made comparisons between RV and LV.

Our comparisons have fulfilled the predictions of an electrophysiological hypothesis involving a reduced *I*_Na_ mechanism. Thus, while the patch-clamp experiments demonstrated that WT myocytes possessed similar peak *I*_Na_ magnitudes in the RV and LV, these were decreased in both the RV and LV in the *Scn5a*+/−, but with a greater reduction in RV than LV. Yet Na^+^ conductance activation curves were indistinguishable. These findings were also compatible with two underlying mechanisms acting upon Na^+^ channel function. First, there could be differing Na^+^ channel inactivation properties in the RV and LV between the WT and the *Scn5a*+/−*.* Thus, although WT RV and LV showed similar inactivation curves, both the RV and LV of the *Scn5a*+/− showed negative shifts in inactivation properties, with the RV showing the greatest alterations. Second, the electrophysiological results implicated reductions in expression of otherwise normal Na^+^ channels. Thus, the Na^+^ current reductions took place even at holding voltages at which there was no significant inactivation. Furthermore, the current reductions exactly paralleled the corresponding patterns of protein expression, corroborating the latter mechanism. So did patterns of mRNA expression, apart from suggesting a relative translational upregulation in the LV relative to the RV *Scn5a*+/−*.* These findings precisely correlated with the outcomes of similar AP (d*V*/d*t*)_max_ values in the RV and LV in the WT, which were reduced in *Scn5a*+/− to a greater extent in the RV than the LV.

Our studies also showed a reduction in *I*_pNa_ in the RV of *Scn5a*+/− hearts, which may well contribute to the shorter APDs in this region. APD values were correspondingly shorter in *Scn5a*+/− RV than WT RV and than *Scn5a*+/− LV. Other groups have reported a gradient of expression of *I*_pNa_ across the ventricular wall [5]. Although the physiological significance in humans is still unclear, the *I*_pNa_ gradient is thought to be important in the pro- and anti-arrhythmic effect of class 1 agents. This is the first study to show heterogeneity of *I*_pNa_ between ventricles in any model, and to contribute to increased repolarization heterogeneity.

Differential K^+^ channel expression has also been implicated in the arrhythmogenic mechanism in BrS. In murine hearts, K^+^ currents include the readily separable non-inactivating *I*_ss_ component. They also include inactivating components—mainly the fast inactivating *I*_to_ and slow inactivating *I*_Ks_. These could be separated through their fast (*τ*_(fast)_) and slow (*τ*_(slow)_) time constants. As described on previous occasions [27,28], *I*_Ks_ appeared to play a relatively minor role in repolarization in the murine ventricle, giving a slow current *I*_slow_ whose magnitude was less than 10 per cent that of the remaining *I*_fast_. Thus, by far the greater part of the outward current observed in the present experiments appeared to arise from *I*_to_.

*I*_to_ channels control a prominent repolarizing current, the Ca^2+^-independent transient outward K^+^ current [34]. While in humans and large mammals, both fast (*I*_to,f_) and slow (*I*_to,s_) channels are expressed throughout the heart, in the mouse heart *I*_to,s_ is present only in the interventricular septum, and thus the focus of this study is on *I*_to,f_, for which the molecular correlates are K_v_4.3/K_v_4.2 [35]. *I*_to,f_ has thus been well documented to be expressed in a heterogeneous manner throughout the ventricles of mammalian hearts, including mice, in concordance with known AP durations [32,36,37]. The present experiments demonstrated larger peak *I*_to_ magnitudes in the RV than the LV. However, there was no particular difference in this peak between *Scn5a*+/− and the WT. Furthermore, there were no differences in the voltage dependence of activation of *I*_to_ in RV or LV whether *Scn5a*+/− or WT. The voltage-dependence of *I*_to_ was slightly shifted to more negative values in the RV than the LV in both WT and *Scn5a*+/−*,* which would potentially reduce its repolarizing effect in parallel with a reduced RV expression of *I*_Na_. However, it was shifted to more positive values in *Scn5a*+/− than WT, which would accentuate the imbalance between repolarizing and depolarizing currents. Furthermore, the RV showed slower inactivation kinetics than did the LV in both WT and *Scn5a*+/−, with a further slowing in the *Scn5a*+/−.

In line with previous studies in mixed ventricular lysates [22], K_ir_2.1 (mediating the inward rectifier *I*_K1_), K_v_1.4 (mediating *I*_to,s_) and K_v_1.5 (mediating *I*_Ks_) demonstrated similar mRNA and protein expression levels. In contrast, K_v_4.2 demonstrated mRNA and protein expression levels greater in the RV than the LV, but with no difference between WT and *Scn5a*+/− in direct correlation with their peak current magnitudes. K_v_4.3 showed a less clear-cut mRNA expression pattern, with small increases in WT RV compared with the remaining groups (in agreement with earlier results), but showed a similar protein expression pattern to K_v_4.2. The disjuncture between current magnitudes and mRNA expression would be consistent with differential KChIP2 expression, in turn controlling expression of the *I*_to_ channel. Our study supports this hypothesis in showing higher mRNA and protein expression of KChIP2 in the RV than LV of WT and *Scn5a*+/− hearts, which could act to increase RV expression of K_v_4.3.

These findings thus directly explain changes in maximum *I*_Na_ and *I*_to_ in terms of altered protein expression. The mechanisms of the altered voltage dependence of the Na^+^ and K^+^ channel inactivation and K^+^ channel current decay particularly in the *Scn5a*+/− RV are unknown, but could possibly be due to changes in binding to regulatory proteins. For example, regulation of Na_v_1.5 is complex, involving interactions with β-subunits and multiple signalling proteins including cytoskeletal molecules such as ankyrins and syntrophins, regulatory kinases and phosphatases, components of cellular trafficking and extracellular matrix molecules, as part of large, multi-protein complexes [38]. Thus, in mice, over-expressing CaMKII, which demonstrates slowed conduction and VT, CaMKII-dependent phosphorylation of Na_v_1.5 shifts the steady-state inactivation curve towards negative voltages in a Ca^2+^-dependent manner [39]. Experiments on neonatal rat ventricles and heterologous cells have demonstrated evidence for functional association of *I*_to,f_ and Na_v_ currents through coupling between Na_v_β1 and KChIP2, leading to co-regulation in normal circumstances [40]. It might be possible that disruption to the coupling mechanism could explain the findings of differential K^+^ channel behaviour in the *Scn5a*+/− hearts. The heterogeneities in *I*_to_ could thus both modify AP repolarization and also influence the reduction of Na^+^ current in *Scn5a*+/− hearts to a different extent in the RV than the LV. This could explain the protective effects of quinidine in reducing the spatial and temporal electrophysiological heterogeneities and incidence of arrhythmias in BrS [41].

The main limitations of our study come from the differences between mouse and human hearts in the repolarization phase of the AP, with differences in the range of K^+^ currents displayed. Mouse hearts also use less L-type Ca^2+^ channel current *I*_Ca,L_*,* with the mouse cardiac AP lacking a plateau phase. The technical difficulty in separating epicardial and endocardial layers meant that our study was limited to a comparison of LV and RV, and we were thus unable to compare transmural differences. Furthermore, we acknowledge that the myocyte isolation procedure is known to cause cell damage, which may affect ionic current properties, including the number of channels and the peak conductance; however, our methodology in the perfusion technique and in the criteria for selection of cells for inclusion in the study aimed to minimize these factors.

Experimental conditions for Na^+^ current measurement were unphysiological, yet chosen for the following reasons. First, voltage-clamp control of the cell membrane was considered. Under physiological conditions, *I*_Na_ is so large and the kinetics are so rapid that successful voltage-clamp control is challenging, even in single-cell preparations [42,43]. For these reasons, we followed protocols that studied *I*_Na_ in a whole-cell recording configuration under reduced experimental temperatures and lower Na*^+^* gradients [16,44–46]. As different temperatures were used for *I*_Na_ measurements from those of *I*_pNa_ and *I*_to_, direct comparisons of these currents cannot be made in this paper. Under our conditions, peak *I*_Na_ was 5.4 ± 0.1 nA and 5.3 ± 0.1 nA for WT LV and RV, respectively; these values are similar to others under similar conditions [16,20,47,48]. To enhance voltage-clamp control, large patch pipettes were used. Thus, peak steady-state voltage error resulting from residual series resistance was 2–3 mV, similar to that found in other studies [16,20,47,49]. If experiments demonstrated evidence of inadequate voltage control (e.g. a ‘threshold phenomenon’ near the voltage range for Na*^+^* channel activation), data were discarded. The absence of crossover of the current traces as the current magnitude was changed and the shape of the I–V relationship remaining unchanged for difference peak current values further suggest adequate voltage control [16,17]. Only around the reversal potential did we find that the shape of the I–V relationship differed slightly between curves, with the WT RV trace giving a slightly more negative reversal potential than would be expected from the calculated Nernst potential of +40 mV. Thus, the clamp voltage was properly controlled in our dialysed cell experiments, and it is unlikely that our findings of reduced *I*_Na_ result from clamp conditions.

The Na^+^ flux recorded may have been affected by voltage-dependent blockage by intracellular Mg^2+^, although this appears to be significant only at high Mg^2+^ concentrations and for outward currents [50,51]. Ca^2+^ flux has also been postulated to occur through Na^+^ channels under β-adrenergic stimulation in a process dubbed ‘slip-mode conductance’ [52], although other groups have found conflicting results [53–55]. While classical studies have shown little affinity of Ca^2+^ or Mg^2+^ for the pore site of the channel [56], the use of low external Na^+^ concentrations may increase possible blockade of the Na^+^ channel by either ion.

While decreased membrane excitability owing to reduced Na^+^ channel function may cause slowed conduction in itself, there is also growing evidence that it may also lead to conduction slowing through the generation of fibrosis [10]. As several previous studies have examined this phenomenon in the *Scn5a*+/− mouse [2], in this study, we chose to focus on the possible electrophysiological mechanisms underlying the arrhythmias; however, we acknowledge the potential roles of fibrosis in both slowing conduction and also in promoting and maintaining differences in repolarization timing between myocardial layers. Thus, both structural changes and differences in ion channel densities and kinetics could together create optimal pro-arrhythmic conditions.

Changes both in *Scn5a*+/− mice and in BrS patients could also involve altered expression of a range of other genes and ion channels. right ventricular outflow tract (RVOT) origins of arrhythmias might also involve slow conducting tissue whose AP upstroke is *I*_Ca,L_-dependent [57]. Ventricular mRNA expression profiles in studies of human BrS associated with Na^+^ channelopathy have suggested remodelled Ca^2+^ in addition to Na^+^ and K^+^ channel expression [58]. Furthermore, Ca^2+^-dependent mechanisms are known to play a role in Na_v_1.5 regulation [59]. Future work might then explore the possibility that altered current densities and expression levels of Ca^2+^ channels might also contribute to arrhythmogenesis in *Scn5a*+/− hearts.

The cellular and molecular experiments described here correlate with work performed in the whole heart, especially mapping experiments that have localized both conduction slowing and heterogeneity of repolarization, and subsequent initiation of arrhythmias through re-entrant phenomena, to the RV [8]. Computer simulations could provide a potential means to further explore the pro-arrhythmic consequences of RV/LV differences in ionic currents, particularly employing whole heart models [60–63]. However, this would also entail obtaining further independent information on detailed conduction pathway morphology, including extents of fibrosis and cell–cell conductance information in *Scn5a*+/− hearts. While groups have previously used whole heart models of WT mouse hearts [60], it has been shown previously that *Scn5a*+/− hearts exhibit heterogeneous fibrosis [2], along with heterogeneous expression of connexin 43 and upregulation of hypertrophic markers including β-MHC and skeletal α-actin [64].

In conclusion, *Scn5a*+/− mice demonstrate a relative upregulation of *Scn5a* expression in the LV compared with RV. The specifically reduced RV expression of Na^+^ channels leads to smaller *I*_Na_, resulting in slowed conduction, and smaller *I*_pNa_, which, in combination with increased *I*_to_, results in shorter AP durations and greater heterogeneity of repolarization, thus suggesting that arrhythmogenesis may be initiated by both abnormal depolarization and repolarization in the RV of *Scn5a*+/− hearts.
